# Antidiabetic Actions of Ethanol Extract of *Camellia Sinensis* Leaf Ameliorates Insulin Secretion, Inhibits the DPP-IV Enzyme, Improves Glucose Tolerance, and Increases Active GLP-1 (7–36) Levels in High-Fat-Diet-Fed Rats

**DOI:** 10.3390/medicines9110056

**Published:** 2022-11-11

**Authors:** Prawej Ansari, J. M. A. Hannan, Samara T. Choudhury, Sara S. Islam, Abdullah Talukder, Veronique Seidel, Yasser H. A. Abdel-Wahab

**Affiliations:** 1 Department of Pharmacy, School of Pharmacy and Public Health, Independent University, Bangladesh (IUB), Dhaka 1229, Bangladesh; 2 School of Biomedical Sciences, Ulster University, Coleraine BT52 1SA, UK; 3 Natural Products Research Laboratory, Strathclyde Institute of Pharmacy and Biomedical Sciences, University of Strathclyde, Glasgow G4 0RE, UK

**Keywords:** hyperglycaemia, glucose, insulin, GLP-1, *Camellia sinensis*, phytoconstituents

## Abstract

*Camellia sinensis* (green tea) is used in traditional medicine to treat a wide range of ailments. In the present study, the insulin-releasing and glucose-lowering effects of the ethanol extract of *Camellia sinensis* (EECS), along with molecular mechanism/s of action, were investigated in vitro and in vivo. The insulin secretion was measured using clonal pancreatic BRIN BD11 β cells, and mouse islets. In vitro models examined the additional glucose-lowering properties of EECS, and 3T3L1 adipocytes were used to assess glucose uptake and insulin action. Non-toxic doses of EECS increased insulin secretion in a concentration-dependent manner, and this regulatory effect was similar to that of glucagon-like peptide 1 (GLP-1). The insulin release was further enhanced when combined with isobutylmethylxanthine (IBMX), tolbutamide or 30 mM KCl, but was decreased in the presence of verapamil, diazoxide and Ca^2+^ chelation. EECS also depolarized the β-cell membrane and elevated intracellular Ca^2+^, suggesting the involvement of a K_ATP_-dependent pathway. Furthermore, EECS increased glucose uptake and insulin action in 3T3-L1 cells and inhibited dipeptidyl peptidase IV (DPP-IV) enzyme activity, starch digestion and protein glycation in vitro. Oral administration of EECS improved glucose tolerance and plasma insulin as well as inhibited plasma DPP-IV and increased active GLP-1 (7–36) levels in high-fat-diet-fed rats. Flavonoids and other phytochemicals present in EECS could be responsible for these effects. Further research on the mechanism of action of EECS compounds could lead to the development of cost-effective treatments for type 2 diabetes.

## 1. Introduction

There has been a significant rise in the demand for herbal medicines in recent years, especially in developing countries, because of their availability, affordability and relative lack of adverse effects compared to conventional drugs. Countries within the Southeast Asian subcontinent have a long history of using plants in traditional medicine to treat a wide range of ailments. The recent use of medicinal plants for the treatment of diabetes has gained prominence [1,2].

Diabetes mellitus (DM), a chronic condition involving defective metabolism of carbohydrates, lipids and proteins, is one of the most prevalent non-communicable diseases around the world [3]. The number of diabetes patients is rising at an alarming rate, with approximately 537 million people currently living with diabetes. This number is expected to exceed 783 million in the next 25 years. DM can be classified into three types—type 1 diabetes mellitus (T1DM), type 2 diabetes mellitus (T2DM) and gestational diabetes, with 10% of DM cases being type 1 and 90% of all cases being type 2 [4,5]. T1DM, which is more prevalent in children and adolescents, is characterized as an autoimmune attack on pancreatic β cells that results in complete deficiency of insulin [5]. T2DM, which is defined as inadequate insulin production, impaired insulin signalling or both, primarily affects individuals over the age of 40 [6]. Insulin resistance, obesity, chronic inflammation, oxidative stress and hyperglycaemia form the basic pathophysiology of T2DM. In addition to these, genetic factors and lifestyle choices also contribute to the prevalence of diabetes [6]. Gestational diabetes is typically diagnosed in women who are at high risk of developing T2DM due to factors such as obesity and physical inactivity [5]. Obesity-associated insulin resistance is a major contributor to the development of T2DM. The excess accumulation of fat in adipose tissues produces non-esterified fatty acids and pro-inflammatory cytokines, which result in insulin resistance and pancreatic β-cell destruction, ultimately leading to T2DM [7]. Obesity also deteriorates type 2 diabetic complications, including cardiovascular diseases (CVDs) and chronic kidney disease (CKD) [8,9].

The main treatment for T2DM includes adherence to a specific diet to control body weight along with synthetic oral drugs. Current therapies for T2DM include different classes of drugs, such as metformin, sulphonylureas, thiazolidinediones, GLP-1 mimetics, DPP-IV inhibitors and sodium-glucose cotransporter-2 (SGLT2) inhibitors [10]. Among these, DPP-IV inhibitors have gained popularity in recent years due to their ability to improve glycaemic control by reducing glucagon release and enhancing insulin secretion [11]. DPP-IV inhibitors work by inactivating the dipeptidyl peptidase IV (DPP-IV) enzyme, which is responsible for suppressing the activity of incretin hormones, glucose-dependent insulinotropic polypeptide (GIP) and glucagon-like peptide-1 (GLP-1) by cleaving in the N terminal and producing GIP (3–42) and GLP-1 (9–36) [12]. Following nutritional consumption, these hormones are produced from the intestine and bind to specific receptors on pancreatic β cells. This interaction promotes insulin secretion from β cells by activating the cyclic adenosine monophosphate (cAMP) pathway. Thus, the use of DPP-IV inhibitors to manage post-prandial hyperglycaemia is a popular approach [13]. However, synthetic DPP-IV inhibitors and other anti-hyperglycaemic agents are often expensive, have limited availability in poorer regions and come with various adverse effects. Alternative approaches to treat T2DM, such as herbal therapy and dietary supplements, have received increased attention recently, especially in lower-income nations [14].

*Camellia sinensis*, commonly known as green tea, is a medicinal plant traditionally used to treat various health conditions, including diabetes, arthritis, bacterial infections and hyperlipidaemia [15]. *C. sinensis* has been demonstrated to decrease total and low-density lipoprotein (LDL) cholesterol as well as triacylglyceride levels, and increase high-density lipoprotein (HDL) cholesterol, thus lowering the risk of developing CVD [16,17]. Previous reports also showed that *C. sinensis* can inhibit DPP-IV enzyme activity and has blood-glucose-lowering and insulin secretory properties [6,18,19,20]. Recent studies have reported that *C. sinensis* contains phytochemicals such as epicatechin, isoquercitrin, rutin, catechin, epicatechin gallate, quercetin, kaempferol, epigallocatechin gallate, ellagic acid, myricetin and gallic acid [6,21,22,23]. Among these, kaempferol, rutin, isoquercitrin, epicatechin, quercetin, gallic acid and catechin have been previously observed to have DPP-IV enzyme inhibitory properties [24,25,26]. Additionally, previous studies have shown that kaempferol, rutin, isoquercitrin, epicatechin, quercetin, ellagic acid and epigallocatechin gallate can lower blood glucose levels, enhance insulin secretion and improve β-cell function [27,28,29,30,31,32,33]. However, there is little information available on the mode of action responsible for the antidiabetic activity of green tea. In this study, in vitro and in vivo methods were used to evaluate the anti-hyperglycaemic effects of the ethanol extract of *C. sinensis* (EECS) leaves and unravel their possible mechanism/s of action.

## 2. Materials and Methods

### 2.1. Collection and Extraction

The leaves of *C. sinensis* were collected from Jahangirnagar University, Dhaka, Bangladesh, authenticated by a botanical taxonomist at Bangladesh National Herbarium (Mirpur, Dhaka) and assigned the accession number 43,207. The leaves were thoroughly rinsed, air-dried and powdered. The plant material (200 g) was soaked in 1 L of 80% (*v*/*v*) ethanol and kept on a shaker at 900 g for 48–72 h at room temperature. Following filtration using filter paper (Whatman no. 1), the extract was dried under reduced pressure at <40 °C to afford a sticky residue that was freeze-dried (Savant Speed vac, New York, NY, USA), and then stored at 4 °C until further studies [34]. 

### 2.2. In Vitro Studies on Insulin Release 

Insulin-secreting clonal pancreatic BRIN-BD11 β cells, generated via electrofusion of primary β cells obtained from rat pancreatic islets (New England Deaconess Hospital) with immortal RINm5F cells [35,36] and isolated mouse islets [37], were employed to assess the insulin secretory effects of the ethanol extract of *C. sinensis* (EECS). Using collagenase P obtained from *Clostridium histolyticum*, islets from mice pancreas were extracted, and cultured in a CO_2_ incubator at 37 °C for 48 h [38]. EECS was incubated in the presence or absence of known insulin secretagogues at 37 °C for 20 and 60 min at various glucose concentrations (5.6 and 16.7 mM) [30]. Samples for the insulin radioimmunoassay were aliquoted and stored at −20 °C until further analysis [39]. To measure the insulin content of the islet cells, an acid–ethanol extraction method was implemented [38].

### 2.3. Membrane Potential and Intracellular Calcium Ions Concentration 

The effects of EECS on the membrane potential and intracellular calcium [Ca^2+^]_i_ in BRIN-BD11 cells were evaluated using a FLIPR Membrane Potential and Calcium Assay Kit (Molecular Devices, Sunnyvale, CA, USA) [33]. BRIN-BD11 cells were seeded into microplates with ninety-six wells and allowed to stand for 18 h in a CO_2_ incubator at 37 °C. A Krebs-Ringer Bicarbonate (KRB) buffer solution (100 μL each well) was added, and the mixture was incubated at 37 °C for 10 min. Depolarizing concentrations of 30 mM KCl and 10 mM alanine were used as the positive controls. A Flex Station 3 fluorometer was used to detect the variations in signal intensity caused by EECS [6]. Variations in signal intensity were detected at excitation, emission and cut-off wavelengths of 530 nm, 565 nm and 550 nm, respectively, for membrane potential and 485 nm, 525 nm and 515 nm, respectively, for intracellular calcium [35].

### 2.4. Assay for Cellular Glucose Uptake

The effects of EECS on cellular glucose uptake were investigated using differentiated 3T3L1 cells [40]. The 3T3-L1 cells were obtained from the American Type Culture Collection (ATCC) (Manassas, VA, USA). Dulbecco’s modified eagle media (DMEM) was made by adding penicillin (50 U/mL), streptomycin (50 μL/mL) and foetal bovine serum (10% *v*/*v*) to DMEM. The differentiated 3T3-L1 cells were incubated with serum-free DMEM for 2 h at 37 °C, at an atmosphere of 5% CO_2_. The DMEM medium was discarded after incubation. The cells were further incubated in Krebs-Ringer Bicarbonate (KRB) buffer for 30 min at 37 °C, in 5% CO_2_ and 95% air. The cells were treated with 50 µL of EECS (200 µg/mL) at 37 °C for 30 min with or without 100 nM insulin, and then 2-[N-(7-nitrobenz-2-oxa-1,3-diazol-4-yl) amino]-2-deoxy-D-glucose (2-NBDG) (50 nM) was added. After allowing the solution to stand for 5 min, the cells were washed with ice-cold PBS. Coverslips were placed over the slides, and the corners were securely sealed. Images of the four corners of the coverslips were captured using a microscope (10× magnification), and the fluorescence intensity was measured as described previously [41].

### 2.5. Insulin Glycation

The effects of EECS on in vitro insulin glycation were assessed as previously described [42]. A solution (1 mL) was prepared by mixing D-glucose (246.5 mM), human insulin (1 mg/mL), sodium phosphate buffer (10 mM, pH 7.4) and NaBH_3_CN (0.0853 gm/mL), with or without EECS (50–200 µg/mL). After 24 h of incubation at 37 °C, the reaction was stopped with 30 µL of 0.5 M acetic acid. The glycated and non-glycated insulin were separated by loading 200 μL of reaction mixture into a (250 × 4.6 mm) Vydac (C-18) analytical column (The Separations Group, California, USA), and then elution was performed at a flow rate of 1 mL/min. The mobile phase consisted of two solvents—solvent A (0.12% (*v*/*v*) TFA/H_2_O) and solvent B (0.1% (*v*/*v*) TFA in 70% acetonitrile + 29.9% H_2_O). To separate the glycated and non-glycated insulin, a linear gradient of 0–35% (*v*/*v*) acetonitrile for 10 min, followed by 35–56% (*v*/*v*) acetonitrile for 20 min and finally 56–70% acetonitrile for 5 min was established. At 214 and 208 nm, elution profiles were detected using RP-HPLC. The insulin glycation inhibitor aminoguanidine was used as a positive control [43]. 

### 2.6. In Vitro Dipeptidyl Peptidase-IV Enzyme Activity

The in vitro dipeptidyl peptidase-IV (DPP-IV) enzyme activity was measured using a fluorometer according to the procedures described previously [44]. Tris-HCl (100 mM) buffer was made by mixing 0.2 M Tris-HCl and 0.1 M NaCl. The pH of this buffer was balanced to 8.0 by adding a 100 mM Tris-base as required. The test reagents, DPP-IV enzyme (8 mU/mL) and Gly-Pro-AMC (200 µM), were dissolved in the buffer and incubated in 96-well black-walled, clear-bottomed microplates (Greiner) with or without EECS (40–5000 µg/mL). The fluorescence intensity was measured using a Flex Station 3 (Molecular Devices, San Jose, CA, USA) with a 2.5 nm slit width and excitation and emission wavelengths of 370 nm and 440 nm, respectively. The standard drug sitagliptin was used as a positive control [34].

### 2.7. In Vitro Digestion of Starch

This in vitro assay was performed to determine the effects of EECS on starch digestion using a previously published protocol [45]. Briefly, heat-stable α-amylase from *Bacillus leicheniformis* (40 µL of 0.01%) (Sigma-Aldrich, St. Louis, MO, USA) was added to a starch solution (2 mg/mL; 100 mg in 50 mL water), with or without EECS (62.5–1000 µg/mL) and incubated at 80 °C for 20 min. The diluted solution was then treated with amyloglucosidase from *Rhizopus* mold (30 µL of 0.1%) (Sigma-Aldrich, St. Louis, MO, USA) at 60 °C for 30 min. The samples were kept at 4 °C until analysis and the glucose release was measured using the liquid glucose oxidase-phenol amino phenazone (GOD/PAP) (Randox GL 2623) method [42]. The α-glucosidase inhibitor acarbose was used as a positive control. 

### 2.8. In Vitro Glucose Diffusion

The in vitro glucose diffusion and absorption was evaluated using a cellulose ester dialysis tube (CEDT) (20 cm × 7.5 mm, Spectra/Por^®^CE layer, MWCO: 2000, Spectrum, Breda, The Netherlands) containing 2 mL of 0.9% NaCl and 220 mM glucose with or without EECS (0.2–25 mg/mL) [35]. The ends were sealed tightly, and the CEDT was placed inside 50 mL Falcon conical tubes (Orange Scientific, Orange, CA, USA) containing 0.9% NaCl (45 mL). Samples were removed from the orbital shaker after 24 h at 37 °C for glucose analysis, as described before [34].

### 2.9. Animals

Sprague Dawley male rats (Envigo UK, around 200–250 g, 6–8 weeks old) were fed a high-fat diet (HFF) (20% protein, 45% fat and 35 % carbohydrate: 26.15 KJ/g total energy percent) (Special Diet Service, Essex, UK) for 6–8 weeks prior to the start of the studies. Age-matched rats fed a standard rodent diet (10% fat, 30% protein and 60% carbohydrate: 12.99 KJ/g total energy) (Trouw Nutrition, Cheshire, UK) were used as normal controls. In total, 108 rats, including normal and high-fat-diet-fed rats, were used in this study. The animals were housed in an environment under controlled temperature and humidity (25 ± 0.5 °C and 65–70 %). The animal housing was equipped with an automatic 12 h light-on/off mechanism that maintained a day–night circadian rhythm. The Ulster University’s Animal Welfare and Ethical Review Board (AWERB) granted approval for experiments to be conducted on animals in May 2018, and the experiments were carried out under project/personal license numbers PIL1822 and PPL 2804 issued by the UK Home Office in May 2016 and February 2017, respectively. All experiments were carried out in compliance with UK Act 1986 and EU Directive 2010/63EU. All precautions were taken to guarantee that no animals would be harmed during the study.

### 2.10. Acute Oral Glucose Tolerance Test 

The effects of EECS on oral glucose tolerance were evaluated in high-fat-diet (HFF)-fed rats. The rats were fasted overnight, and blood samples were collected using tail vein bleeding. Blood samples were taken at specific time intervals prior to (0 min) and after (30, 60, 120 and 180 min) oral administration of glucose (18 mmol/kg body weight) with/without EECS (250 mg/5 mL/kg). After centrifuging the blood for 5 min at 12,000 rpm at 4°C, the plasma was collected and kept at −20 °C until the insulin assay was performed. Blood glucose levels were monitored using Ascencia Contour glucose meters (Bayer, Newbury, UK), and insulin levels were assessed using a dextran–charcoal radioimmunoassay [46]. 

### 2.11. In Vivo Dipeptidyl Peptidase-IV Enzyme Activity

The effects of EECS on DPP-IV enzyme activity were evaluated in the plasma of animals using a fluorometric assay [30]. HFF rats were fasted overnight, and blood samples were obtained at a specific time interval before (0 min) and after (30, 60, 120 and 180) oral administration of EECS (250 mg/5 mL/kg), DPP-IV inhibitors sitagliptin (10 µmoL/5 mL/kg) and vildagliptin (10 µmoL/5 mL/kg) or saline control. Centrifugation was performed to collect plasma serum. In 96-well microplates, plasma samples (10 μL) were incubated with 40 μL of Tris-HCl (100 mM) buffer (pH 7.4) and 50 μL of Gly-Pro-AMC (200 μM) substrate at 37 °C for 30 min. When the DPP-IV enzyme in the blood serum hydrolyzed the fluorogenic substrate bonds (H-Gly-Pro) that were conjugated to the AMC group (H-Gly-Pro-AMC), the fluorescent 7-Amino-4-Methyl Coumarin (AMC) was produced. FlexStation 3 was used to measure the fluorescence changes as stated above in the in vitro DPP-IV enzyme activity section. Active GLP-1 (7–36) levels were quantified in plasma samples obtained at 30 min using a GLP-1 (Active) ELISA Kit (EGLP-35K, Merck Millipore, Dorset, UK).

### 2.12. Feeding Test 

The effects of EECS on food intake were observed in HFF rats. Rats were starved for 12 h before the experiment was carried out. The food intake was measured at 0, 30, 60, 90, 120, 150 and 180 min before or after oral administration of saline (5 mL/kg), EECS (250 and 500 mg/5 mL/kg) and glibenclamide (100 mg/5 mL/kg), respectively. The standard drug glibenclamide was used as a positive control.

### 2.13. Metabolic Studies 

Metabolic studies in HFF rats were performed using metabolic cages to measure food and fluid intake, stools and urine. The rats underwent a 24 h adaption period followed by a 12 h fasting period. EECS (250 and 500 mg/5 mL/kg) was fed to the treatment groups while the positive control group was fed glibenclamide (100 mg/5 mL/kg), and the HFF control group was given saline (5 mL/kg) individually. The food and fluid intake of each group was measured and the amount of stool and urine excreted out recorded. These four factors were measured at 1 h intervals over a period of 6 h initially, followed by 2 h intervals over the next 6 h and then after 24 h.

### 2.14. Gut Motility

Gastrointestinal motility was measured using a BaSO_4_ milk solution (10% BaSO_4_ *w*/*v* in 0.5% Na-CMC), as previously described [47]. Rats were starved for 20 h. One hour before consuming the BaSO_4_ solution, the treatment groups received EECS (250 and 500 mg/5 mL/kg), bisacodyl (10 mg/5 mL/kg) and loperamide (5 mg/5 mL/kg). The animals were euthanized 15 min after receiving the BaSO4 milk solution, and the whole intestine was isolated. The distance travelled by BaSO4 was measured and calculated as a percentage of a total length of the small intestine (from the pylorus to the ileocecal junction).

### 2.15. Statistical Analysis

Graph Pad prism 5 was used to analyze and interpret the raw data. The unpaired Student’s t-test (non-parametric, with two-tailed *p* values) and one-way ANOVA with Bonferroni post hoc tests were used to analyze the data. All values are expressed as mean ± SEM with a hypothetical statistical significance limit of *p* < 0.05. 

### 2.16. Phytochemical Screening

Phytochemical screening of EECS was conducted to demonstrate the presence or absence of flavonoids, alkaloids, saponins, tannins, glycosides, reducing sugar and steroids, as described previously [48]. To test for alkaloids, 2 mL of EECS was acidified using hydrochloric acid (HCl), and 1 mL of Dragendroff’s reagent was added to test for the appearance of a red colour, suggesting the presence of alkaloids. For tannins, a few drops of 10% lead acetate were added to 2 mL of EECS, and this caused the formation of a white sediment, suggesting the presence of tannins. Flavonoid testing was conducted by adding 1.5 mL of methanol to 4 mL of EECS and then heating this mixture; when metal magnesium and a few drops of HCL were added to this, a pink colour appeared, indicating the presence of flavonoids. To test for saponins, 1 mL of EECS was added to 9 mL of distilled water, and this resulted in the formation of a stable foam, indicating the presence of saponins. To test for steroids, 2 mL of EECS was mixed with 10 mL of chloroform, 1 mL of acetic anhydride and 2 mL of sulphuric acid to visualize a bluish-green colour, which indicates the presence of steroids. For glycoside testing, 1 mL of EECS was mixed with a few drops of glacial acetic acid, ferric chloride and concentrated sulphuric acid to test for the appearance of a bluish-green colour, indicating the presence of glycosides. To test for reducing sugar, 1 mL of EECS, 1 mL of water and few drops of Fehling’s reagent were mixed together and heated, and we looked for the appearance of a red-brick colour indicating the presence of reducing sugars [48].

## 3. Results

### 3.1. EECS and Insulin Release from BRIN BD11 Cells

**Figure 1:**
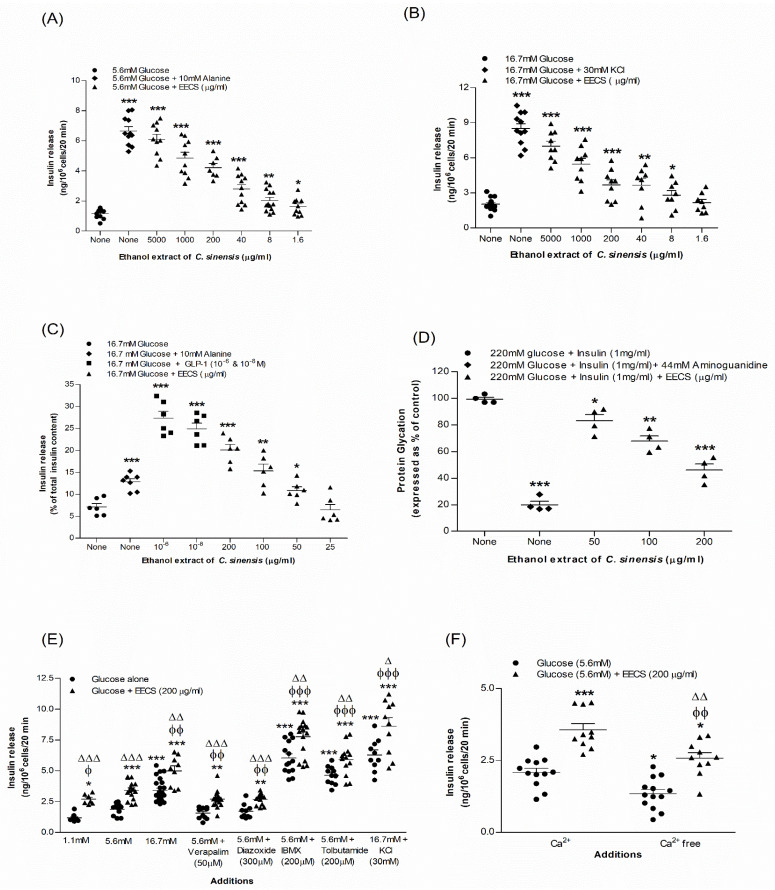
Effects of EECS on insulin secretion from (**A**,**B**) clonal pancreatic BRIN-BD11 β cells and (**C**) islets of Langerhans, (**D**) glycation of protein (**E**), secretion of insulin with known stimulators/inhibitors and (**F**) plus/minus extracellular calcium from BRIN-BD11 cells. Values *n* = 4–8 for insulin secretion and glycation of protein are mean ± SEM. *^,^ **^,^ *** *p* < 0.05–0.001 compared to control. ^ϕ, ϕϕ, ϕϕϕ^ *p* < 0.05–0.001 compared to 5.6 mM glucose with EECS. ^Δ, ΔΔ, ΔΔΔ^ *p* < 0.05–0.001 compared to respective incubation without EECS. EECS, ethanol extract of *C. sinensis*.

Figure 1A,B illustrate the effects of EECS on insulin release from BRIN-BD11 cells in a concentration (1.6–5000 µg/mL)-dependent manner. At 5.6 mM glucose, the basal rate of insulin release from BRIN-BD11 cells was 1.16 ± 0.10 ng/10^6^ cells/20 min. Using alanine (10 mM) as a positive control, the rate increased to 6.65 ± 0.29 ng/10^6^ cells/20 min (Figure 1A; *p* < 0.001). At 5.6 mM glucose, EECS stimulated insulin release from 1.62 ± 0.18 to 6.09 ± 0.34 ng/10^6^ cells/20 min (Figure 1A; *p* < 0.05–0.001) in a dose-dependent manner (1.6–5000 µg/mL). The basal insulin rate at 16.7 mM glucose was 2.04 ± 0.14 ng/10^6^ cells/20 min and with the positive control, KCl (30 mM), the rate was increased to 8.50 ± 0.42 ng/10^6^ cells/20 min (Figure 1B; *p* < 0.001). At 16.7 mM glucose, EECS induced insulin release from 2.80 ± 0.41 to 7.02 ± 0.44 ng/10^6^ cells/20 min (Figure 1B; *p* < 0.05–0.001) at 8–5000 µg/mL. No significant effect of EECS on lactate dehydrogenase release was found at concentrations ranging from 1.6 to 200 µg/mL.

### 3.2. EECS and Insulin Release from Isolated Mouse Islets

Figure 1C shows the effects of EECS on insulin release from the isolated mouse islets. At 16.7 mM glucose, the basal rate of insulin release from isolated mouse islets was 7.15 ± 0.78 ng/10^6^ cells/20 min (Figure 1C). EECS stimulated insulin release from 10.85 ± 0.88 to 20.12 ± 1.24 ng/10^6^ cells/20 min (Figure 1C; *p* < 0.05–0.001) in a concentration-dependent manner (50–200 µg/mL). The positive controls alanine (10 mM) and GLP-1 (10^−6^ and 10^−8^ M) showed a significant (*p* < 0.001) increase in insulin release from 12.85 ± 0.70 to 27.35 ± 1.55 (Figure 1C). However, in comparison to alanine, the insulin release caused by GLP-1 was more potent (*p* < 0.001).

### 3.3. EECS and Known Modulators/Inhibitors of Insulin Release

EECS significantly enhanced insulin release in the presence of insulin modulators, including glucose (*p* < 0.001), isobutylmethylxanthine (IBMX; *p* < 0.001) and tolbutamide (*p* < 0.001) (Figure 1E). In the presence of a depolarizing concentration of KCl, EECS also induced a substantial increase in insulin release (*p* < 0.001; Figure 1E). Diazoxide, verapamil and Ca^2+^-free conditions attenuated, but did not completely abolish, this effect (*p* < 0.01; Figure 1E,F). 

### 3.4. EECS and Cell Membrane Depolarization and [Ca^2+^]_i_ Concentration

**Figure 2:**
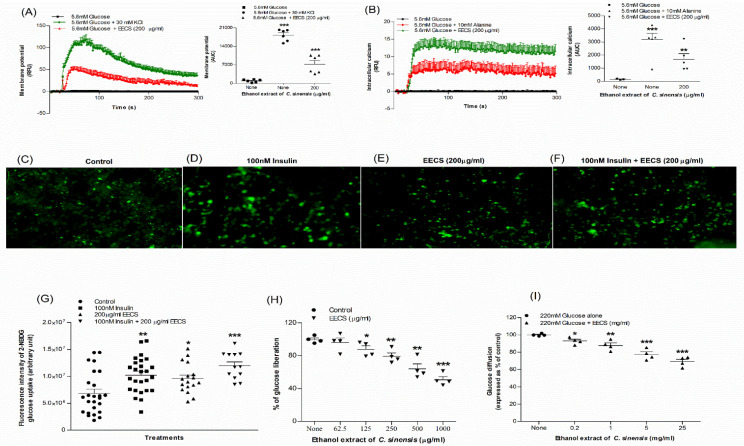
Effects of EECS on (**A**) membrane potential and (**B**) intracellular calcium in clonal pancreatic BRIN BD11 β cells and (**C**–**G**) glucose uptake, (**H**) starch digestion and (**I**) glucose diffusion in vitro. Changes in fluorescence intensity in differentiated 3T3L1 adipocyte incubated with EECS (**E**) minus or (**F**) plus 100 nM insulin. Magnification of 10x was used to capture the images. Values *n* = 6 for membrane potential and intracellular calcium, *n* = 4 for uptake of glucose, digestion of starch and diffusion of glucose are mean ± SEM. *, **, *** *p* < 0.05–0.001 compared to control.

The effects of EECS on membrane potential and intracellular calcium concentrations ([ Ca^2+^]_i_) were evaluated using BRIN-BD11 cells (Figure 2A,B). At a concentration of 200 μg/mL, EECS significantly depolarized the cell membrane (*p* < 0.001; Figure 2A) and increased intracellular calcium ion concentration (*p* < 0.001; Figure 2B). The positive controls KCl (30 mM) and alanine (10 mM) showed a greater response on the membrane potential (*p* < 0.001; Figure 2A) and intracellular calcium (*p* < 0.001; Figure 2B), respectively.

### 3.5. EECS and Insulin Glycation

The effects of EECS on insulin glycation are depicted in Figure 1D. EECS inhibited insulin glycation by 16–53% (*p* < 0.05–0.001; Figure 1D) at 50–200 µg/mL, whereas the positive control aminoguanidine (44 mM) suppressed insulin glycation by 82% (*p* < 0.001; Figure 1D). 

### 3.6. EECS and Glucose Uptake

The uptake of glucose in 3T3L1 adipocyte cells was assessed using fluorescent 2-NBDG (2-(N-(7-Nitrobenz-2-oxazol-4-yl) Amino)-2-Deoxyglucose). The microscopic fluorescence intensity of 2-NBDG uptake is shown in Figure 2C–F. In differentiated 3T3L1 adipocyte cells, EECS (200 μg/mL) improved glucose uptake with/without 100 nM insulin (*p <* 0.05; *p <* 0.001; Figure 2G). Glucose uptake was also considerably enhanced by 100 nM insulin alone (*p <* 0.01; Figure 2G).

### 3.7. EECS and Starch Digestion

EECS decreased starch digestion by 12–54% (*p* < 0.05–0.001; Figure 2H) in a dose-dependent manner (125–1000 µg/mL). The positive control acarbose (1000 µg/mL) inhibited starch digestion by 79% (data not shown). 

### 3.8. EECS and In Vitro Glucose Diffusion 

The effects of EECS on glucose diffusion following a 24 h incubation with glucose are shown in Figure 2I. EECS (0.2–25 mg/mL) significantly decreased glucose diffusion/absorption by 7–27% (*p* < 0.05–0.001; Figure 2I) in a concentration-dependent manner. 

### 3.9. EECS and In Vitro Dipeptidyl Peptidase-IV Enzyme Activity

**Figure 3:**
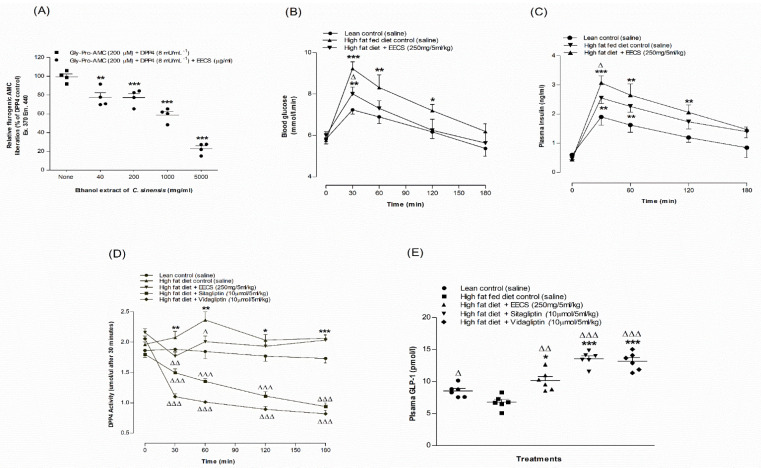
Effects of EECS on (**A**) DPP-IV enzyme in vitro, (**B**) glucose tolerance, (**C**) plasma insulin, (**D**) DPP-IV and (**E**) active GLP-1 (7–36) in high-fat-diet-fed rats. In vivo parameters were evaluated before and after oral administration of glucose alone (18 mmol/kg body weight, control) or with EECS (250 mg/5 mL/kg body weight), sitagliptin and vidagliptin (both at 10 μmol/5 mL/kg, body weight). Plasma active GLP-1 (7–36) levels were measured 30 min following treatment. Values *n* = 4 for in vitro DPP-IV enzyme activity and *n* = 6 for in vivo parameters are mean ± SEM. *, **, *** *p* < 0.05–0.001 compared to control and ^Δ, ΔΔ, ΔΔΔ^ *p* < 0.05–0.001 compared to high-fat-diet-fed control rats.

The effects of EECS on in vitro DPP-IV enzyme activity are illustrated in Figure 3A. In the presence of the DPP-IV enzyme, EECS (40–5000 μg/mL) showed a significant (*p <* 0.05–0.001) decrease (16–72%) in AMC liberation from Gly-Pro-AMC (Figure 3A). The DPP-IV inhibitor sitagliptin significantly (*p <* 0.001) attenuated (98%) AMC liberation from Gly-Pro-AMC (data not shown).

### 3.10. EECS and Oral Glucose Tolerance and Plasma Insulin Levels 

Oral gavage of EECS (250 mg/5 mL/kg) in combination with glucose (18 mmoL/5 mL/kg body weight) significantly improved oral glucose tolerance at 30 and 60 min in HFF rats (*p* < 0.05; Figure 3B) compared to the control. EECS (250 mg/5 mL/kg) also significantly ameliorated plasma insulin levels at 30 min in HFF rats (*p* < 0.05; Figure 3C). 

### 3.11. EECS and Plasma DPP-IV Enzyme Activity and Active GLP-1 (7–36) Levels

Oral gavage of EECS (250 mg/ 5 mL/kg, body weight) significantly decreased plasma DPP-IV enzyme activity at 30 and 60 min (*p* < 0.05–0.01; Figure 3D) compared to HFF rats. Interestingly, there was a consistent reduction in plasma DPP-IV enzyme activity in the presence of sitagliptin (10 µmoL/5 mL/kg) and vidagliptin (10 µmoL/5 mL/kg) in a time-dependent manner (*p* < 0.001; Figure 3D). Oral administration of EECS (250 mg/5 mL/kg body weight) elevated plasma active GLP-1 (7–36) levels in the circulation by 32% (*p* < 0.01; Figure 3E), and this was increased to 82–90% (*p* < 0.001; Figure 3E) with sitagliptin (10 µmoL/5 mL/kg) and vidagliptin (10 µmoL/5 mL/kg), respectively. 

### 3.12. EECS and Feeding Test

**Figure 4:**
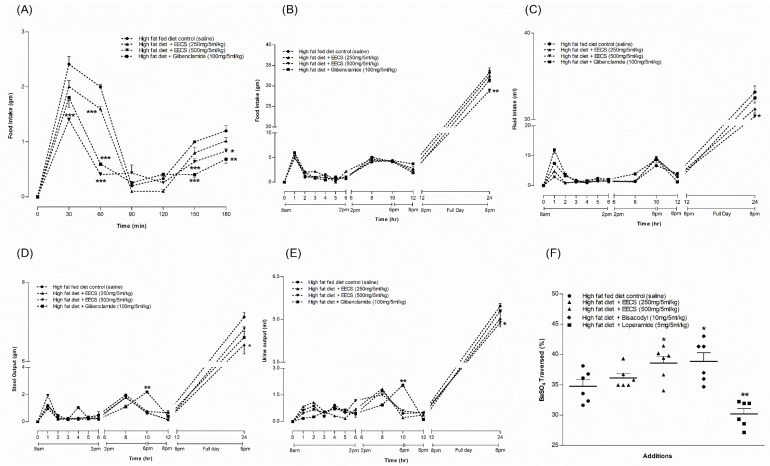
Effects of EECS on (**A**) food intake during feeding test and (**B**) food intake, (**C**) fluid intake, (**D**) stool and (**E**) urine output after 24 h of metabolic study. (**F**) BaSO_4_ traversed. Values *n* = 6 for feeding test and metabolic parameters are mean ± SEM. *, **, *** *p* < 0.05–0.001 compared to high-fat-diet-fed control rats.

EECS, at 500 mg/5 mL/kg, consistently reduced the food intake at most of the time points (*p <* 0.05; *p <* 0.001; Figure 4A), whereas at 250 mg/5 mL/kg, it significantly decreased the food intake only at 30 and 60 min (*p <* 0.001; Figure 4A). The sulfonylurea glibenclamide also substantially decreased the food intake in a time-dependent manner (*p <* 0.01–0.001; Figure 4A).

### 3.13. EECS and Metabolic Parameters

EECS (250 and 500 mg/5 mL/kg, b.w.) decreased food consumption at night (between 6 and 8 pm) and after 24 h (*p <* 0.01; Figure 4B) compared to HFF rats alone. EECS (250 and 500 mg/5 mL/kg, b.w.) also reduced fluid intake at night (between 6 pm and 8 pm) and after 24 h (*p <* 0.05; Figure 4C) compared to HFF rats alone. EECS also attenuated stool and urine output at night (between 6 and 8 pm) and after 24 h (*p <* 0.05; Figure 4D,E), which was consistent with food and fluid intake. The positive control, glibenclamide, improved these parameters in comparison to HFF rats alone (Figure 4B–E). Glibenclamide also significantly improved the frequency of stool and urine output at night (between 6 and 8 pm; *p <* 0.01; Figure 4D,E). 

### 3.14. EECS and Gastrointestinal Motility

EECS (500 mg/5 mL/kg) significantly improved gastrointestinal motility (*p <* 0.05; Figure 4F). However, at 250 mg/5 mL/kg, it showed no significant improvement in gut motility (Figure 4F). The antidiarrheal drug loperamide (5 mg/5 mL/kg) decreased gut motility ((*p <* 0.01; Figure 4F), while the stimulant laxative bisacodyl (10 mg/5 mL/kg) increased gut motility (*p <* 0.05; Figure 4F). 

### 3.15. EECS and Phytochemical Screening

To establish the presence of possible antidiabetic phytochemicals, further investigation was carried out. EECS was found to contain alkaloids, flavonoids, saponins and tannins (Table 1).

## 4. Discussion

Diabetes is one of the most widespread and devastating metabolic illnesses in the world, affecting millions of people [49]. Although T2DM can be managed using oral anti-hyperglycaemic agents, it often requires the use of synthetic insulin in the long term. Many oral antidiabetic drugs, such as sulfonylureas, biguanides, glinides, glycosidase and DPP-4 inhibitors, have adverse side-effects and/or are expensive. Moreover, the long-term use of insulin increases insulin receptor sensitivity and causes insulin resistance [35,50]. Natural products have become an important source of safer and more economical anti-hyperglycaemic drugs. Medicinal plants and their constituents (e.g., flavonoids) have been reported previously to exhibit antidiabetic properties, including insulin-releasing and glucose-lowering activities, inhibiting α-amylase and α-glucosidase and protecting and improving the function clonal pancreatic β cells [51,52]. The regular consumption of dietary fibres from plants has also been reported to reduce the incidence of diabetes [53]. 

*Camellia sinensis* has been reported to possess remarkable pharmacological activities in traditional medicine against various ailments including diabetes [54]. It was recently reported to have antidiabetic properties, and previous studies have demonstrated that *C. sinensis* lowers blood glucose levels, improves glucose tolerance and prevents hyperlipidaemia by reducing total cholesterol and LDL levels in diabetic animal models [55,56,57]. However, the exact mechanism of action of *Camellia sinensis* remains elusive. Our results reveal that EECS enhanced insulin secretion in a concentration-dependent manner from BRIN-BD11 cells and isolated mouse islets in response to glucose stimulation. Specific secretory pathways were targeted using insulin-releasing/inhibiting modulators to develop a better understanding of the mechanism of action of a non-toxic dose of EECS on β cells [58]. In the presence of the K_ATP_ channel opener diazoxide, the insulin-releasing activity of EECS was reduced, suggesting that *C. sinensis* might act via K_ATP_, a channel-dependent pathway [59]. The voltage-dependent calcium channel blocker verapamil also reduced the insulin release mediated by EECS. These findings suggest that the mode of action of EECS involves the closing of K_ATP_ channels and the opening of L-type Ca^2+^ channels [60]. EECS also increased insulin release in the presence of the depolarizing concentration of KCl (30 mM) and the K_ATP_ channel blocker tolbutamide. These results support the ability of EECS to potentiate insulin secretion via other pathways, such as a direct effect on exocytosis, the phosphatidylinositol (PI3) or the adenylate cyclase/cAMP pathways [61]. The cAMP phosphodiesterase inhibitor IBMX also potentiated the insulin secretion induced by EECS, indicating a modulation of intracellular cAMP production [41]. Thus, EECS may be involved in the increase in cAMP levels in lung muscle tissue and the relaxation of smooth muscles in the airway passage [62].

Post-prandial glucose is controlled by insulin via glucose transporter 4 (GLUT4) translocation in skeletal muscle and adipose tissue [63]. Inadequate or defective signalling reduces GLUT4 translocation and leads to the development of insulin resistance [64]. In this study, we investigated the effect of EECS on glucose uptake in differentiated 3T3L1 adipocyte cells. EECS enhanced glucose uptake in the presence and absence of insulin. Previous studies have revealed that phytochemicals such as kaempferol, quercetin and gallic acid could activate the AMP-activated protein kinase (AMPK) pathway and enhance GLUT4 translocation [65,66]. The fact that *C. sinensis* is known to contain flavonoids, such as rutin, isoquercitrin and catechin, may explain how it can activate signalling pathways to enhance glucose transport in adipocytes with or without insulin [6,67,68].

The increased glycation of insulin has a vital role in the pathogenesis of numerous disease, such as diabetes, leading to the formation of advanced glycation end products (AGEs). AGEs accumulate in cells, resulting in impaired cell signalling, and increasing the severity of diabetes complications [69]. EECS was found to decrease insulin glycation in a dose-dependent manner. Phytochemicals isolated from *C. sinensis*, such as epigallocatechin gallate, isoquercitrin and rutin [6,70], have previously been observed to have anti-glycating properties [71,72,73]. Our results suggest that *C. sinensis* possesses potent anti-glycating activity. This is possibly due to the presence of polyphenolic compounds.

Numerous factors are involved in the pathophysiology of diabetes, including starch digestion by α-amylase and α-glucosidase and glucose absorption and diffusion in the gastrointestinal tract [73]. EECS significantly decreased starch digestion in a concentration-dependent manner. We hypothesize that rutin and isoquercitrin, known to be present in C. *sinensis*, might be responsible for such activity [74]. Previous studies showed that these flavonoids are effective against α-amylase and delay starch digestion [75]. EECS also demonstrated significant concentration-dependent inhibition of glucose absorption and diffusion, which is consistent with previous studies on a hot water extract of *C. sinensis* [6].

Obesity, a major risk factor of T2DM, is characterized by the presence of non-esterified fatty acids (NEFAs) released from adipose tissue that contribute to insulin resistance and β-cell dysfunction, causing T2DM [76]. In our study, EECS ameliorated oral glucose tolerance and plasma insulin levels in HFF obese rats. This is consistent with previous results showing that *C. sinensis* improves oral glucose tolerance, plasma insulin and β-cell function in HFF rats [6,77] and streptozotocin (STZ)-induced diabetic rats [48]. 

Our further in vivo studies using HFF rats showed that EECS reduced plasma DPP-IV enzyme activity, which is consistent with our in vitro results. EECS also enhanced active GLP-1 (7–36) levels in the circulation. GLP-1, an incretin hormone secreted by the intestine after a meal, plays a crucial role in maintaining post-prandial glucose homeostasis [78]. Thus, GLP-1 mimetics and DPP-IV inhibitors are important targets in T2DM drug discovery research. Interestingly, previous studies have revealed that flavonoids found in *C. sinensis*, such as rutin and isoquercitrin, have DPP-IV enzyme inhibitory activity [74,79,80].

Feeding test and metabolic studies were performed to observe the effects of EECS on different parameters, including food and fluid intake, as well as stool and urine output. EECS was found to reduce all of the four abovementioned parameters, specifically at night between 6 pm and 8 pm. Rats are most active at night and diabetic rats usually have the highest blood glucose levels at the night phase of their circadian rhythm [81]. The blood-glucose-lowering activity of EECS may be due to a reduction in food intake during that time period. 

Phytochemical screening of EECS showed the presence of phytoconstituents including flavonoids, alkaloids, tannins and saponins. Recent reports also showed that *C. sinensis* consists of flavonoids such as rutin and isoquercitrin [67,68,82] and alkaloids [83], as well as tannins and saponins [84]. Flavonoids have previously been found to inhibit in vitro α-glucosidase activity, as well improve glucose tolerance, enhance insulin release and protect pancreatic β cells from oxidative stress damage in HFF- and STZ-induced diabetic rats [6,85,86,87]. In addition, alkaloids and saponins are known to regulate glucose homeostasis via the AMPK pathway; along with this, tannins have been observed to increase glucose uptake via the phosphatidylinositol (PI3) pathway [88,89,90]. The presence of these phytochemicals may have contributed to the insulin-releasing and glucose-lowering effects of EECS. Further investigations, particularly long-term animal studies, are required to fully understand the role of EECS in T2DM.

## 5. Conclusions

Our results suggest that EECS may increase the insulin secretion ability of clonal pancreatic β cells. EECS impeded glucose diffusion and absorption, insulin glycation and DPP-IV enzyme activity in vitro. In our in vivo studies*,* EECS improved glucose tolerance and plasma insulin levels, and decreased plasma DPP-IV enzymatic activity while elevating active GLP-1 levels in the circulation, showing that EECS may be involved in the augmentation of the GLP-1 and GIP half-life. The presence of flavonoids in *C. sinensis*, such as rutin and isoquercitrin, may be responsible for the insulin-releasing and glucose-lowering effects observed for EECS. Our findings validate, to a certain extent, the traditional use of *Camellia sinensis* as a dietary supplement for T2DM. Further studies, including the purification and identification of active compounds from EECS, may help researchers to discover new drug templates for the management of T2DM.

## Figures and Tables

**Table 1:** Phytochemical screening of ethanol extract of *C. sinensis*.

Group Test	Observation
Alkaloids	*+*
Tannins	*+*
Saponins	*+*
Steroids	*-*
Glycoside	*-*
Flavonoids	*+*
Reducing Sugar	*-*

(***+***) = present, (***-***) = absent.

## Data Availability

Due to certain restrictions, the information is unavailable to the public. The corresponding author can disclose the data obtained from this study upon request.
